# Male and female are not the same: a multicenter study of static and dynamic functional connectivity in relapse-remitting multiple sclerosis in China

**DOI:** 10.3389/fimmu.2023.1216310

**Published:** 2023-10-10

**Authors:** Yao Wang, Yunyun Duan, Yuling Wu, Zhizheng Zhuo, Ningnannan Zhang, Xuemei Han, Chun Zeng, Xiaoya Chen, Muhua Huang, Yanyan Zhu, Haiqing Li, Guanmei Cao, Jie Sun, Yongmei Li, Fuqing Zhou, Yuxin Li

**Affiliations:** ^1^ Department of Radiology, The First Affiliated Hospital, Nanchang University, Nanchang, Jiangxi, China; ^2^ Clinical Research Center For Medical Imaging In Jiangxi Province, Nanchang, Jiangxi, China; ^3^ Department of Radiology, Beijing Tiantan Hospital, Capital Medical University, Beijing, China; ^4^ Department of Radiology and Tianjin Key Laboratory of Functional Imaging, Tianjin Medical University General Hospital, Tianjin, China; ^5^ Department of Neurology, China-Japan Union Hospital of Jilin University, Changchun, Jilin, China; ^6^ Department of Radiology, The First Affiliated Hospital of Chongqing Medical University, Chongqing, China; ^7^ Department of Radiology, Huashan Hospital, Fudan University, Shanghai, China

**Keywords:** relapsing-remitting multiple sclerosis, magnetic resonance imaging, sex, independent component analysis, static functional network connectivity, dynamic functional network connectivity

## Abstract

**Background:**

Sex-related effects have been observed in relapsing-remitting multiple sclerosis (RRMS), but their impact on functional networks remains unclear.

**Objective:**

To investigate the sex-related differences in connectivity strength and time variability within large-scale networks in RRMS.

**Methods:**

This is a multi-center retrospective study. A total of 208 RRMS patients (135 females; 37.55 ± 11.47 years old) and 228 healthy controls (123 females; 36.94 ± 12.17 years old) were included. All participants underwent clinical and MRI assessments. Independent component analysis was used to extract resting-state networks (RSNs). We assessed the connectivity strength using spatial maps (SMs) and static functional network connectivity (sFNC), evaluated temporal properties and dynamic functional network connectivity (dFNC) patterns of RSNs using dFNC, and investigated their associations with structural damage or clinical variables.

**Results:**

For static connectivity, only male RRMS patients displayed decreased SMs in the attention network and reduced sFNC between the sensorimotor network and visual or frontoparietal networks compared with healthy controls [*P*<0.05, false discovery rate (FDR) corrected]. For dynamic connectivity, three recurring states were identified for all participants: State 1 (sparse connected state; 42%), State 2 (middle-high connected state; 36%), and State 3 (high connected state; 16%). dFNC analyses suggested that altered temporal properties and dFNC patterns only occurred in females: female patients showed a higher fractional time (*P*<0.001) and more dwell time in State 1 (*P*<0.001) with higher transitions (*P*=0.004) compared with healthy females. Receiver operating characteristic curves revealed that the fraction time and mean dwell time of State 1 could significantly distinguish female patients from controls (area under the curve: 0.838-0.896). In addition, female patients with RRMS also mainly showed decreased dFNC in all states, particularly within cognitive networks such as the default mode, frontoparietal, and visual networks compared with healthy females (*P* < 0.05, FDR corrected).

**Conclusion:**

Our results observed alterations in connectivity strength only in male patients and time variability in female patients, suggesting that sex-related effects may play an important role in the functional impairment and reorganization of RRMS.

## Introduction

Multiple sclerosis (MS) is an immune-mediated demyelinating disease of the central nervous system (CNS) and is a leading non-traumatic cause of disability in young adults (1). Previous studies have reported that female MS patients have a higher incidence of recurrences (2–4), but male patients with MS seem to have more severe physical disability and to progress faster (5, 6). There is also strong evidence indicating that sex plays a crucial role in the recurrence and progression of MS. However, the underlying mechanisms of these sex-related effects within the CNS are complex and have not been fully studied in MS. Structural MRI studies found that male patients with MS seems to have a higher lesion load (7, 8), more severe microstructural damage, such as atrophy of gray matter and/or deep gray matter (9, 10), and demyelination of white matter (11), although these remain understudied.

In addition to structural damage, sex-related functional reorganization also appears to exist in relapsing-remitting MS (RRMS). Recently, static disconnectivity and network efficiency decreases of the default mode network were found in male RRMS patients and related to impaired visuospatial memory (12). However, only increased static functional connectivity was found in male RRMS patients from another study, but this sex-related difference of functional connectivity was no longer significant after regressing gray matter volume (13). These conflicting findings may result from sex-related differences in structural damage and functional reorganization of the CNS in RRMS patients with different sex.

Furthermore, recent research has shown that brain networks are not truly “static” but instead change over time during MRI scans (14). Dynamic functional network connectivity (dFNC) enables quantification of the connectivity strength and its temporal properties of dynamic changes on very short time scales (15). A growing number of studies have found that dFNC can identify recurring dynamic connectivity states in different subtypes and disease stages of MS and decreased dynamic functional connectivity was strong association with cognitive impairment (16, 17), fatigue (18), and disability (19). However, it remains unclear whether sex affects the dynamic connectivity of functional networks in RRMS patients.

As such, our study hypothesized that the connectivity strength and time variability of functional networks in RRMS patients are also affected by sex and related to brain structural damage or physical disability. To test this hypothesis, we retrospectively analyzed 208 patients with RRMS (135 females/73 males) and 228 healthy controls (123 females/105 males) from six centers in China, evaluating the spatial distribution strength, static functional connectivity strength, and dFNC pattern and its temporal properties of the resting-state networks (RSNs) in RRMS. Our study may reveal the pathophysiological mechanism of neurological damage in large-scale functional networks in RRMS patients by different sex.

## Materials and methods

### Standard protocol approvals and patient consents

All subjects signed written informed consent forms, and this study was approved by the local ethics review board at each center.

### Subjects

This retrospective multicenter study recruited 593 subjects (including 263 MS and 331 healthy controls) from six centers in China (from 2009 to 2019). All subjects needed to be right-handed, 18 to 65 years old, undergo MRI scans, and have complete clinical information. All MS patients were diagnosed by the 2017 Revised McDonald Criteria as being in the RRMS category. The Expanded Disability Status Scale (EDSS) was used to evaluate the patients’ overall disability. Ultimately, 135 female patients with RRMS (RRMS females) and 73 male patients with RRMS (RRMS males), matched for age, disease duration (DD), and EDSS scores, and 123 healthy females and 105 healthy males were recruited as controls.

### MRI acquisition

All participants underwent 3.0 T MRI scans; the required scan sequences included high-resolution 3D T1-weight, T2-weight, fluid-attenuated inversion recovery (FLAIR), and resting-state functional MRI (rs-fMRI). The specific scan parameters of each center are described in our previous study (20).

### Structure measurement

White matter volume (WMV) and gray matter volume (GMV) were segmented and calculated automatically using the tissue probability maps method in the computational anatomy toolbox (CAT12). In addition, the ratio of brain parenchymal tissue (GMV plus WMV) to total intracranial cavity volume was defined as the brain parenchymal fraction (BPF).

As described in our previous study (20), lesion volume (LV) was manually delineated and checked by 5- and 11-year experienced radiologists based on the T2-weighted or FLAIR images, and lesion masks were created. The lesion masks were transformed into the Montreal Neurological Institute (MNI) space and the lesion volume calculated in the SPM12 platform.

### Resting-state fMRI data preprocessing

fMRI data were preprocessed using the Resting-State fMRI Data Analysis Toolkit plus (REST plus v1.25) package based on SPM12 (Statistical Parametric Mapping) and MATLAB v8.40 (The Mathworks, Inc., U.S.). Since the scanning duration to acquire rs-fMRI data may be different in each center, fMRI data preprocessing was performed separately for each center (20). The processing pipeline included: 1) discarding the first 10 image volumes; 2) head movement realignment, the mean framewise displacement (FD) of each subject was evaluated to reflect mean head movement; 3) spatial normalization into the Montreal Neurological Institute (MNI) space and resampling with 3 × 3 × 3 mm^3^; and 4) spatial smoothing (full width at half maximum (FWHM)=6 mm).

### Independent component analysis (ICA)

**Figure 1:**
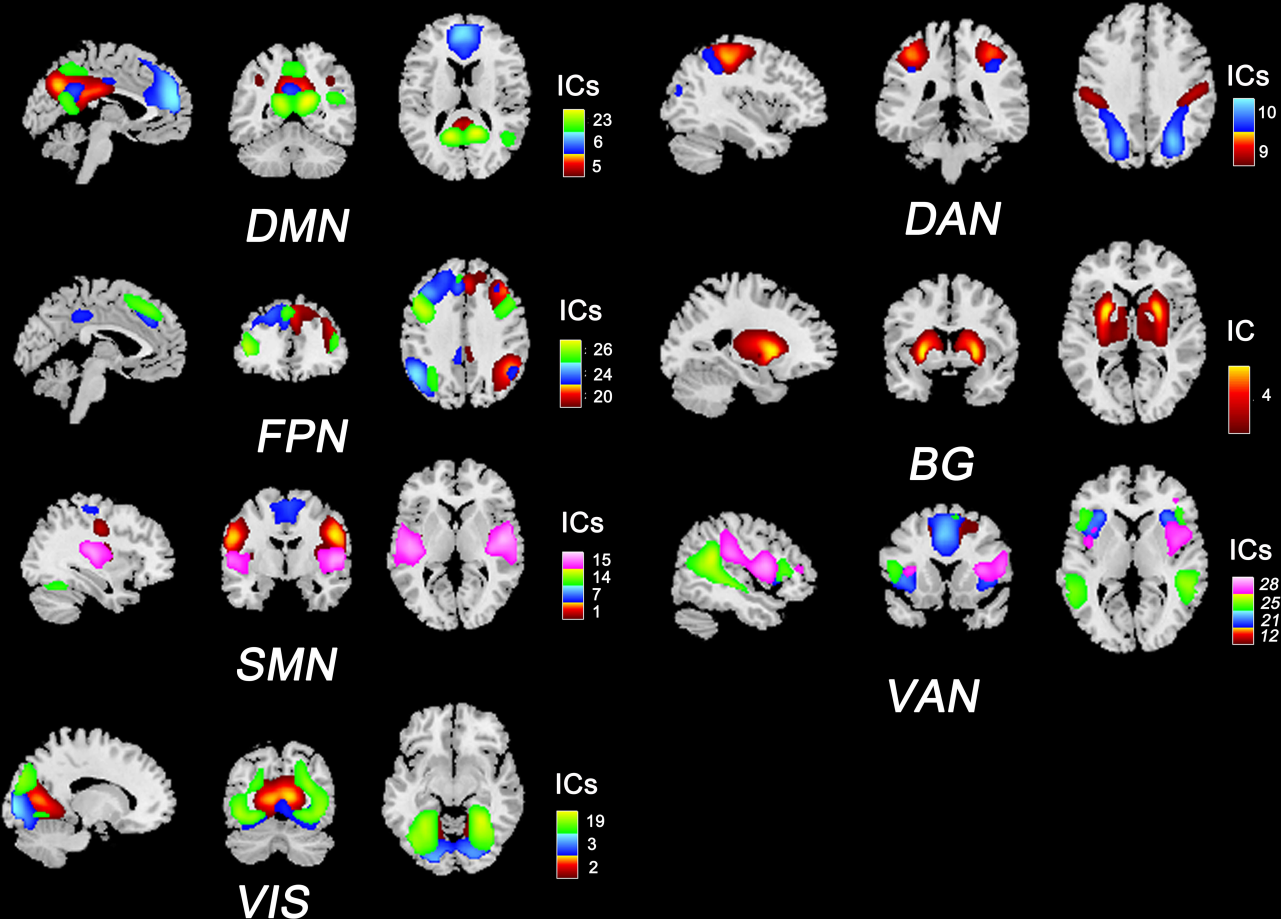
Composite maps of 20 independent components and seven RSNs. The number and color of each component correspond to the color bars. DMN, default mode network; SMN, sensorimotor network; VIS, visual network; FPN, frontoparietal network; DAN: dorsal attention network; VAN, ventral attention network; BG, basal ganglia network.

We used ICA to identify RSNs rather than using seed-based approaches because ICA is data-driven and does not require prior assumptions (e.g., selecting the seed regions). In this study, we implemented spatial ICA to extract temporally coherent and spatially independent sources within the fMRI time course using the Group ICA Of fMRI Toolbox (GIFT v3.0b). The pipeline included: 1) dimensionality reduction with principal component analysis; 2) evaluating components using the Infomax algorithm and ICASSO algorithm (100 iterations); and 3) back reconstruction using the GICA3 algorithm. According to the works of Allen et al. (21) and Yeo et al. (22), 20 independent components (ICs) and seven RSNs were then obtained for further analysis: the default mode network (DMN), the sensorimotor network (SMN), the visual network (VIS), the frontoparietal network (FPN), the dorsal attention network (DAN), the ventral attention network (VAN), and the basal ganglia network (BG). The composite maps and peak coordinates of the ICs and RSNs are shown in **Figure 1** and **Table S1**.

### Static functional network analysis

Multivariate analysis of covariance (MANCOVAN) was applied to assess the significant associations between the spatial map and static connectivity strength of the RSNs and group status (RRMS females, RRMS males, healthy females, and healthy males). We used univariate tests and regressed the age, mean FD, and multicenter variables as covariates to evaluate the effects of disease and sex.

### Intra-network spatial maps

To assess the intra-network connectivity strength, SMs were thresholded based on the distribution of voxelwise T statistics (i.e., mean ± 4SD) to evaluate the consistent and highly activated voxels within each network. This procedure requires an individual t-test for each voxel within a spatial map with a false discovery rate (FDR) correction at *P*<0.05.

### Inter-network static functional network connectivity

For inter-network sFNC, we selected the default options to perform postprocessing on subject-specific time courses, including detrending using 3dDespike and filtering using a fifth-order Butterworth low-pass filter with a high-frequency cutoff of 0.15 Hz. Pearson’s correlations were computed for each pair of components and sFNC matrices were obtained for all subjects.

### Dynamic functional network analysis

The dFNC was carried out in the Temporal dFNC toolbox in GIFT. A sliding window approach was used with a window width of 22 repetition times (TRs) (44s), a step of 1 TR (2s), and a Gaussian convolution of 3 TRs (6s). Furthermore, using L1 regularized inverse covariance matrix (repeated 10 times) was used to disperse the dFNC matrix, and Fisher’s Z-transformation was applied. Then, K-means clustering with Manhattan distance (150 iterations and five repetitions) was performed to distinguish recurring dFNC patterns within different windows for each subject. According to the elbow criterion, the optimal number of clusters was three or four (k= 3 or 4). The dFNC temporal properties included: 1) fraction time (the total time percentage of one subject staying in a state); 2) mean dwell time (the time each subject spent in a specific state); and 3) transition number (the total number of transitions from one state to another).

### Control for head movement

The following approaches were to reduce the potential effects of head motion on sFNC and dFNC: 1) subjects with translation > 3 mm or rotation > 3° were excluded; 2) ICA was used to identify and remove motion-related components from the fMRI data (23); 3) rs-fMRI time courses were detrended using 3dDespike and filtered using a fifth-order Butterworth low-pass filter with a high-frequency cutoff of 0.15 Hz; 4) the mean FD was regressed in the ANOVA tests of sFNC and dFNC between groups; and 5) six motion-realignment parameters were regressed on the sFNC and dFNC matrix for each subject.

### Statistical analysis

**Figure 2:**
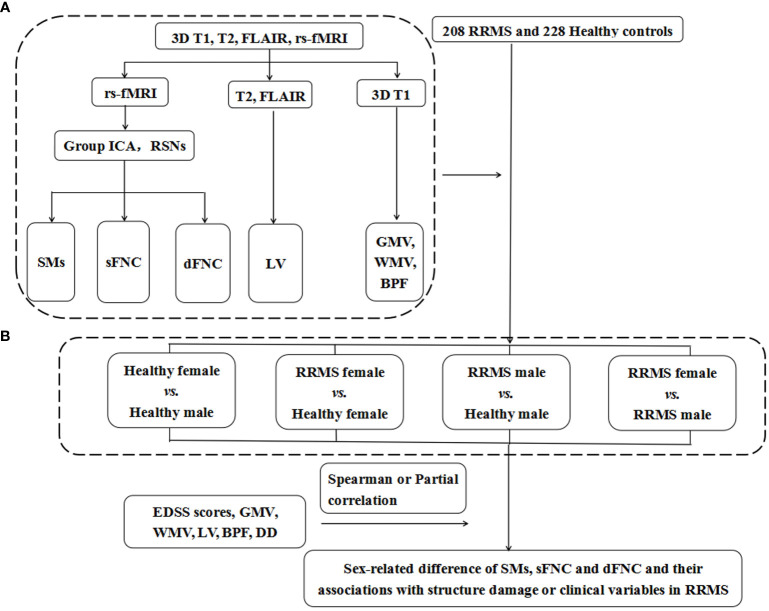
The flow chart of Materials and Methods. **(A)** The data of 3DT1, T2, rs-fMRI, and FLAIR images were preprocessed and postprocessed to calculate functional and structural indicators for each subject, including the SMs, sFNC, and dFNC and LV, GMV, WMV, and BPF. **(B)** A total of 208 RRMS patients and 228 healthy controls were recruited for this study, and they were divided into four groups according to sex. We compared the differences in SMs, sFNC, and dFNC between the above four groups to determine the sex effects in patients with RRMS. RRMS, relapsing-remitting multiple sclerosis; rs-fMRI, resting-state functional MRI; FLAIR, fluid-attenuated inversion recovery; Group ICA, group independent component analysis; RSNs, resting-state networks; SMs, spatial maps; sFNC, static functional network connectivity; dFNC, dynamic functional network connectivity; GMV, gray matter volume; WMV, white matter volume; BPF, brain parenchyma fraction; LV, lesion volume; DD, disease duration; EDSS score, Extended Disability Status Scale score.

**Figure 2** shows the flow chart of the Methods. The demographic data, lesion volume, and other clinical variables were analyzed in SPSS 23.0. We applied Kolmogorov–Smirnov tests to evaluate the normality of the clinical data and ANOVA or Mann–Whitney U tests for differences between groups. One-way ANOVA and *post hoc* tests (*P*<0.05) with FDR correction were performed to evaluate significant associations between the SM, sFNC, and dFNC of the RSNs and group status: 1) healthy female vs. healthy male; 2) RRMS female vs. healthy female; 3) RRMS male vs. healthy male; and 4) RRMS female vs. RRMS male. Moreover, we used the Mann–Whitney U test (*P*<0.01) to compare the temporal features of the dFNC between groups. Receiver operating characteristic (ROC) curves analysis assessed the performance of static and dynamic indicators in distinguishing RRMS from healthy controls. Spearman or partial correlation analysis was applied to explore the relationships between altered functional or structural measures and clinical variables. The ANOVA and correlation analysis were applied with age, mean FD, and multi-center variables as covariates or control variables. Moreover, to investigate the effect of gray matter volume on functional networks, we also supplemented the ANOVA analysis with gray matter volume as a confounding factor between groups.

### Reproducibility

The choice of window width for a sliding window method is a matter of debate. Previous studies have suggested that a window width between 30 s and 60 s could extract physiological signals (24, 25) and was not affected by noise. Thus, we also added the results of a window width of 30 TRs and the number of clusters of three or four (k=3 or 4).

### Data availability

Correspondence and requests for the data can be addressed to our corresponding author, Fuqing Zhou.

## Results

**Table 1** shows all demographic data and group statistics. Lower GMV, WMV, and BPF were shown in RRMS patients compared with controls (P ≤ 0.002). Male RRMS patients showed higher GMV and WMV than female patients (P<0.001), but there was no statistically significant difference in BPF between female and male RRMS patients (P=0.423). Among the patients with RRMS, male patients had a higher lesion load (*P*=0.041) than female patients, even though the recruited RRMS patients were matched between sexes for age, disease course, and clinical disability. There was no significant difference in age or mean FD between the RRMS groups and healthy controls (*P*: 0.101-0.730).

**Table 1:** Demographic data and clinical characteristics.

	RRMS (n=208)		Healthy controls (n=228)		*P* value			
	Female (n=135)	Male (n=73)	Female (n=123)	Male (n=105)	*P1*	*P2*	*P3*	*P4*
Age (years)^a^	37.55 (11.47)	35.42 (10.98)	36.94 (12.17)	38.17 (10.87)	0.405	0.682	0.101	0.192
DD (months)^b^	17 (5–48)	24 (5–72)	–	–	–	–	–	0.492
Mean FD (mm)^a^	0.068 (0.107)	0.078 (0.117)	0.069 (0.105)	0.075 (0.119)	0.225	0.730	0.475	0.113
EDSS scores^b^	2 (1-3.5)	2.5 (1.5-3.5)	–	–	–	–	–	0.309
LV (ml)^b^	5.94 (1.43-16.70)	8.16 (3.36-19.70)	–	–	–	–	–	0.041
GMV (ml)^a^	604.20 (58.58)	656.31 (61.14)	653.84 (57.84)	687.42 (64.19)	<0.001	<0.001	0.002	<0.001
WMV (ml)^a^	453.51 (56.39)	512.96 (72.18)	498.03 (51.67)	549.67 (51.93)	<0.001	<0.001	<0.001	<0.001
BPF^a^	0.76 (0.05)	0.75 (0.04)	0.80 (0.03)	0.79 (0.03)	0.019	<0.001	<0.001	0.423

^a^indicates data are presented as the mean (standard deviation); ^b^indicates data are presented as the median (interquartile range).

*P1*: Healthy females vs. healthy males.

*P2*: RRMS females vs. healthy females.

*P3*: RRMS males vs. healthy males.

*P4*: RRMS females vs. RRMS males.

RRMS, relapsing-remitting multiple sclerosis; FD, framewise displacement; DD, disease duration; EDSS, Extended Disability Status Scale; LV, lesion volume; GMV, gray matter volume; WMV, white matter volume; BPF, brain parenchyma fraction.

### Static functional network analysis

#### Intra-network SMs and inter-network sFNC

**Figure 3:**
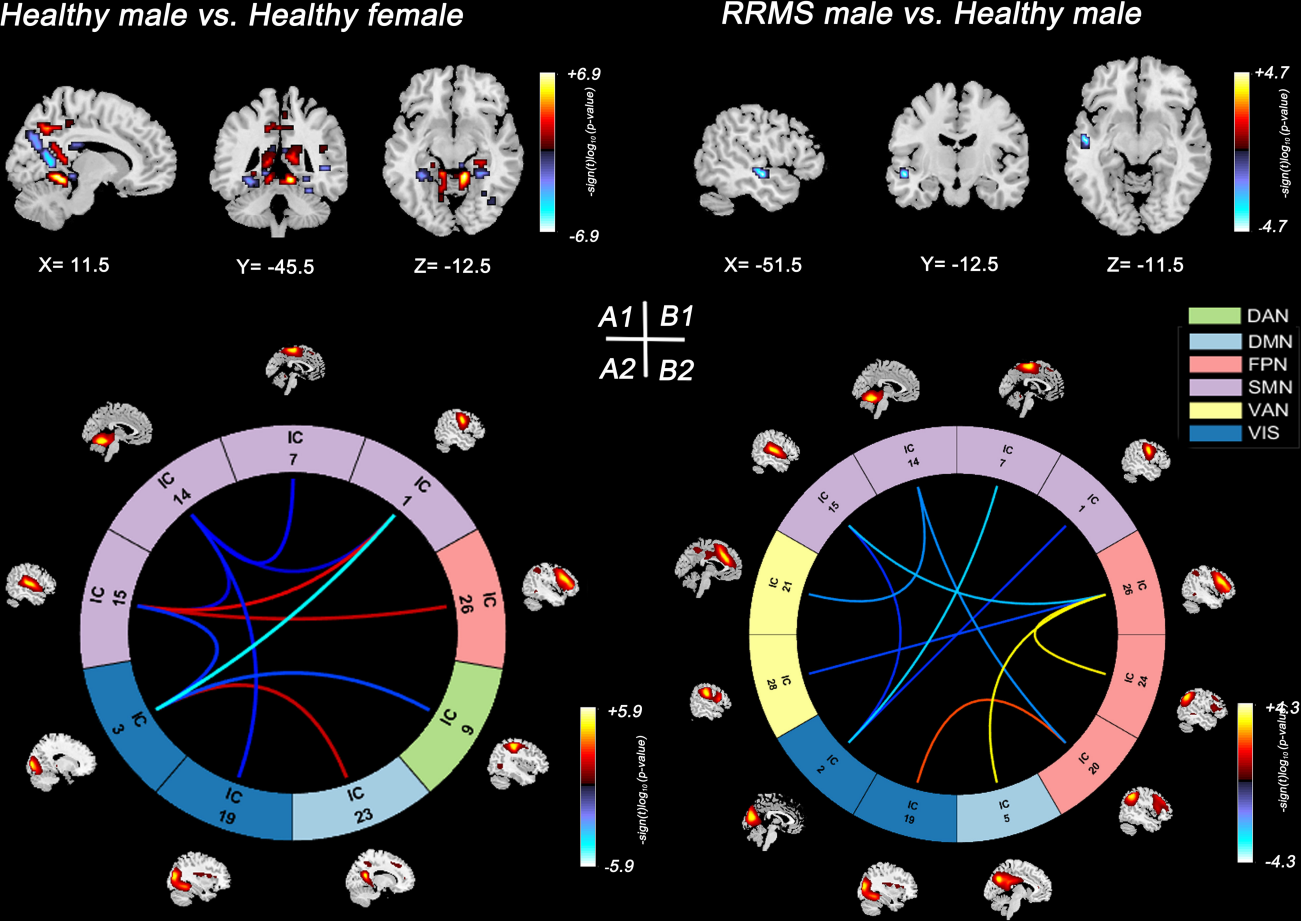
Significant results of the voxel-level comparison of RSN maps and sFNC between groups. Spatial maps of significant voxels **(A1)** and sFNC **(A2)** of RSNs in healthy groups, *P*<0.05, FDR corrected. Spatial maps of significant voxels **(B1)** and sFNC **(B2)** of RSNs between RRMS males and healthy males, *P*<0.05, FDR corrected. Significant cluster volume>10. RRMS, relapsing-remitting multiple sclerosis; DMN, default mode network; SMN, sensorimotor network; VIS, visual network; FPN, frontoparietal network; DAN, dorsal attention network; VAN, ventral attention network.

In healthy controls, healthy males showed lower SMs within the DMN (bilateral precuneus) and lower sFNC within the SMN and SMN-VIS compared with healthy females (*P*<0.05, FDR corrected) (**Figures 3A1, A2**). However, this sex difference disappeared between female and male patients with RRMS. Compared with healthy controls, only RRMS males exhibited decreased SMs within DAN, increased sFNC within PFN, and reduced sFNC of SMN-PFN, SMN-VIS, and SMN-VAN (*P*<0.05, FDR corrected) (**Figures 3B1, B2**). There was no significance in RRMS females vs. healthy females and RRMS females vs. RRMS males.

#### Dynamic functional network analysis

##### dFNC clustering states

**Figure 4:**
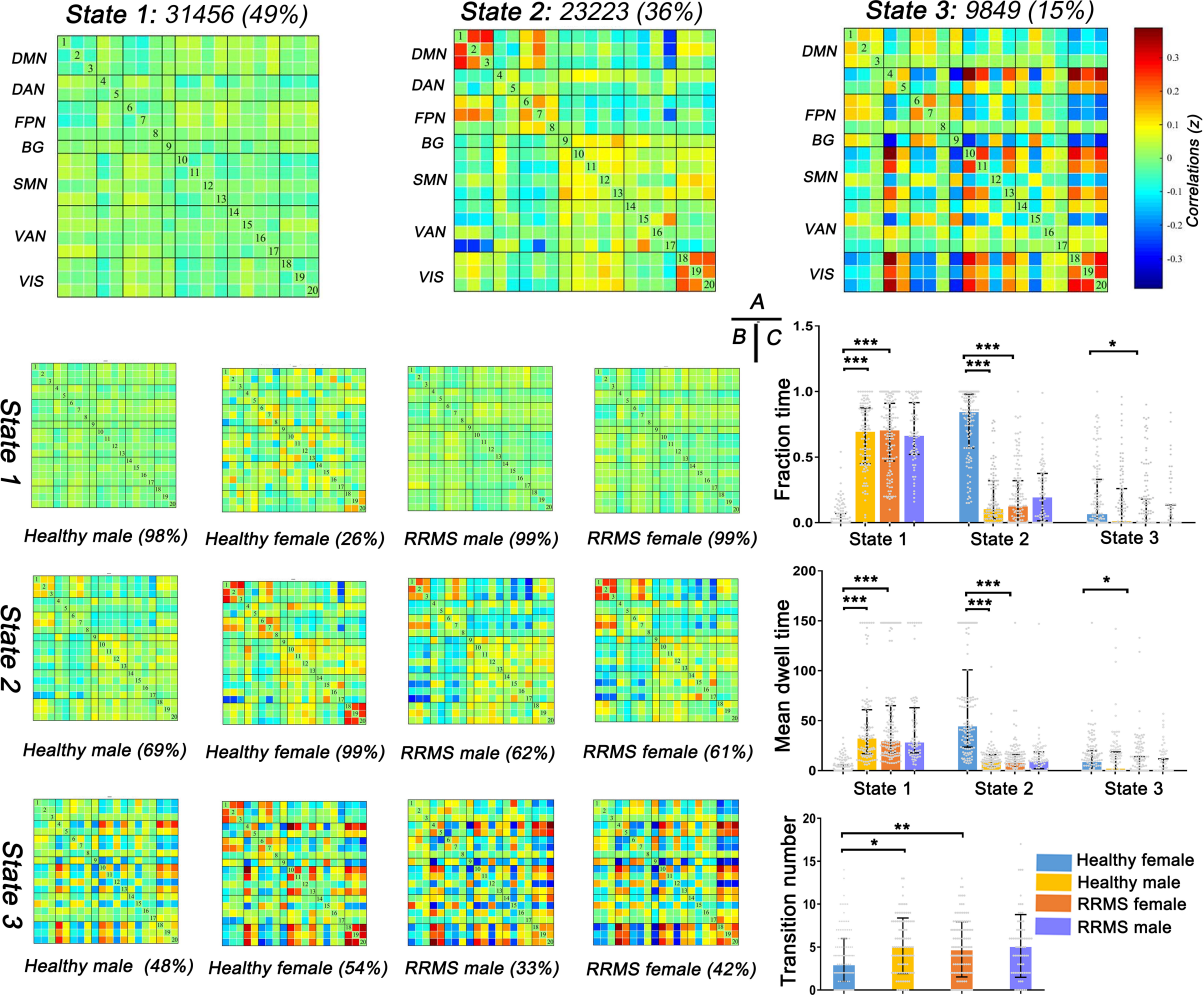
Results of clustering analysis and between-groups dynamic temporal properties. **(A)** Three dFNC states for all subjects with the total number of occurrences and percentage of total occurrences. **(B)** The specific connectivity state for each group. The percentage is calculated as the ratio of the number of subjects who entered one state to the total number of subjects in each group. **(C)** Significant differences in the temporal properties in healthy controls and RRMS groups: healthy females showed a higher fraction time and more dwell time in State 2 (*P*<0.001) with lower transitions (*P*=0.012) compared with healthy males, while female RRMS patients exhibited a higher fraction time and more dwell time in State 1 (*P*<0.001) and higher transitions (*P*=0.004) relative to healthy females. Fraction time: the total time percentage of one subject staying in a state. Mean dwell time: the time each subject spent in a specific state. Transition number: the total number of subject transitions from one state to another. *** indicates *P*<0.001, ** means *P*<0.01, * means *P*<0.05. RRMS, relapsing-remitting multiple sclerosis; DMN, default mode network; SMN, sensorimotor network; VIS, visual network; FPN, frontoparietal network; DAN, dorsal attention network; VAN, ventral attention network; BG, basal ganglia network.

First, three recurrent dFNC states were identified after cluster analysis: State 1 (sparse connected state; 49%), State 2 (middle connected state; 36%), and State 3 (high connected state; 15%) (**Figure 4A**). State 1 was characterized by sparse connectivity both within and between networks, whereas State 3 showed a tightly connected matrix. State 2 was a transitional state between State 1 and State 3, which featured decreased dFNC between the SMN and DMN or FPN and increased dFNC within the DMN, FPN, SMN, and VIS. **Figure 4B** shows the percentage of specific states for each group: healthy females showed higher percentage in State 2 (99%), whereas healthy males and RRMS patients showed higher percentage in State 1 (98%, 99%,99%, respectively).

##### dFNC temporal properties

Next, the temporal properties of dFNC revealed significant differences in fraction time, mean dwell time, and transitions among the three states among the groups (**Figure 4C**). Compared with healthy males, healthy females showed a higher fraction time (healthy female vs. healthy male: 85% vs. 11%; *P*<0.001) and more dwell time in State 2 (healthy female vs. healthy male: 45.33s vs. 4.42s; *P*<0.001) with lower transitions (healthy female vs. healthy male: three times vs. five times; *P*=0.035). However, the tendency changed once RRMS was established; female RRMS patients exhibited a higher fraction time (RRMS female vs. healthy female: 71% vs. 0%; *P*<0.001) and more dwell time in State 1 (RRMS female vs. healthy female: 30.25s vs. 0.00s; *P*<0.001) and higher transitions (RRMS female vs. healthy female: four times vs. three times; *P*=0.004), relative to healthy females. No statistical difference was seen in RRMS males vs. healthy males and RRMS females vs. RRMS males. Furthermore, ROC curve analysis found that the fraction time and mean dwell time of State 1 could significantly distinguish female patients from controls [areas under the curve (AUC): 0.838, 0.896, respectively] (**Data Sheet 1**, **Figure S1**).

##### Between-group dFNC differences

**Figure 5:**
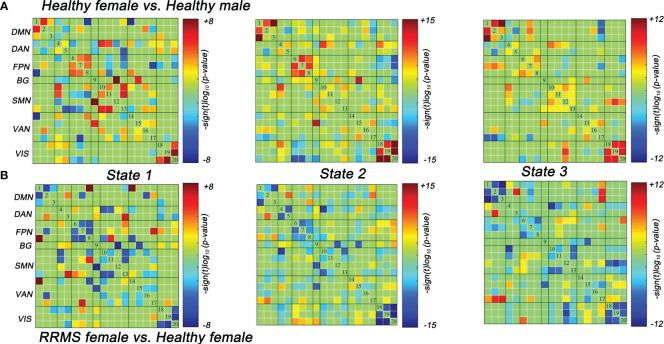
Significant differences in dFNC between groups. **(A)** Healthy females showed significantly higher dFNC within DMN, PFN, and VIS compared with healthy males (*P*<0.05, FDR corrected). **(B)** RRMS females showed significantly lower dFNC within DMN, PFN, and VIS compared with healthy females (*P*<0.05, FDR corrected). RRMS, relapsing-remitting multiple sclerosis; DMN, default mode network; SMN, sensorimotor network; VIS, visual network; FPN, frontoparietal network; DAN, dorsal attention network; VAN, ventral attention network; BG, basal ganglia network.

Lastly, we also evaluated the dFNC differences between the healthy controls and RRMS groups. Similar to the results of the dFNC temporal properties, the dFNC pattern alterations were seen only in female groups: compared with healthy males, healthy females exhibited higher dFNC within DMN, FPN, and VIS in all states (*P*<0.05, FDR corrected) (**Figure 5A**). On the contrary, this trend disappeared among RRMS patients. Moreover, compared with healthy females, female patients mainly showed lower dFNC in all states, particularly within the DMN, FPN, and VIS (*P*<0.05, FDR corrected) (**Figure 5B**). There was no dynamic significance in RRMS males vs. healthy males and RRMS females vs. RRMS males.

We next analyzed the between-group differences in functional network with gray matter volume as a covariate, and we found that gray matter volume had no significant effect on the results of SMs, sFNC and dFNC between RRMS groups and healthy controls. For more detailed results (including the description), see **Data Sheet 2**, **Figures S2**–**S4**.

##### Correlation analysis

**Figure 6:**
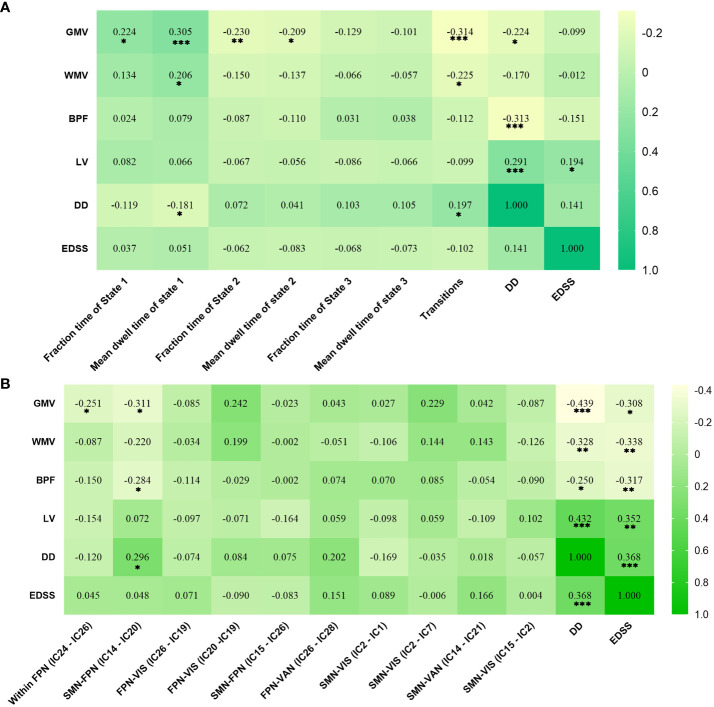
Significant correlation analysis results of RRMS patients. **(A)** Correlation results in female RRMS patients. **(B)** Correlation results in male RRMS patients. ***indicates P<0.001, **means P<0.01, *means P<0.05. Abbreviations: RRMS, relapsing-remitting multiple sclerosis; GMV, gray matter volume; WMV, white matter volume; BPF, brain parenchyma fraction; LV, Lesion volume, EDSS, extended disability status scale; DD, disease duration.

In RRMS females, the lower fraction time (*r* = 0.224, *P* = 0.011) and mean dwell time (*r* = 0.305, *P* < 0.001) of State 1 related to lower GMV, and the higher numbers of transitions related to lower GMV (*r*=-0.314, *P*<0.001) and lower WMV (*r*=-0.225, *P*=0.011). For RRMS males, decreased sFNC of SMN-FPN was related to lower GMV (*r*=-0.311, *P*=0.012) and BPF (*r*=−0.284, *P*=0.022). The correlations with significant p values (*P*<0.05) are shown in **Figure 6**, **Tables S2**, **S3**.

##### Reproducibility

In these replication analyses, when the window width was 30 TRs or 22 TRs, and the clustering state was 3 or 4, we found that RRMS affected dFNC patterns and its temporal properties only in female patients with RRMS, indicating the dFNC results were very stable, reliable, and repeatable. The details are as follows:

The differences of temporal properties between groups suggested that RRMS females preferred State 1 (sparse connected state) and spent more time in State 1 compared with healthy females (*P*<0.001) (**Figures S5**–**S7**).

The dFNC differences between groups suggested that RRMS females showed decreased dFNC within DMN, FPN, and VIS in all states compared with healthy females (*P*<0.05, FDR corrected) (**Figures S8**–**S10**).

## Discussion

Our study investigated the between-group differences in the strength and time-varying connectivity of brain networks between RRMS and healthy subjects by sex and the associations with structural damage and disability. sFNC alterations were only observed in male patients with increased sFNC within the FPN and decreased sFNC across the SMN-FPN and SMN-VIS networks, whereas dFNC abnormalities were observed only in female RRMS patients, manifested as a higher fraction time and more dwell time in State 1 (sparse connected state) with lower transitions compared with healthy females. A higher fraction time and more dwell time of State 1 could significantly distinguish female patients from controls. Altered sFNC and altered temporal properties were related to structural damage in RRMS patients.

### Significant sFNC alterations in male patients with RRMS

Given the disease damage to the brain, it is easy to understand that RRMS patients have lower gray matter and white matter volumes compared with healthy controls, and atrophy of gray and white matter has been reported in MS (26). However, RRMS males still had a higher lesion burden compared with female patients, even though the recruited RRMS patients were matched for age, disease duration, and clinical disability between sexes. This is consistent with previous studies that male patients seem to have a higher level of brain structural damage (9, 13). Further analysis of sFNC revealed increased and reduced sFNC in only male patients with RRMS compared with controls, mainly decreased sFNC across the SMN-FPN and SMN-VIS networks; these results remained after regressing gray matter volume. Our findings could be understood by the “network collapse”, a widely accepted model for explaining sFNC changes in MS proposing that sFNC change is a dynamic process and network efficiency tends to deteriorate with subsequent declines in sFNC, due to progressive brain structural damage and the reduced compensatory capacity of functional networks in MS (27). Furthermore, these functional networks have been widely reported in MS, and functional reorganization of the SMN occurs in all stages of relapse, remission, and recovery in RRMS, and are closely related to clinical disability (28, 29). Furthermore, functional changes in the FPN and VIS are involved in cognitive dysfunctions in RRMS patients, including attention (30), working memory (31), and visual information processing (32).

We analyzed the correlation analysis between structural measures and connectivity changes and found that there was no correlation in statistics between lesion load and altered connectivity in both female and male patient cohorts. These results may indicate that the sFNC reorganization in MS patients may be caused by multiple factors, such as damage of gray matter and white matter (33, 34). Indeed, our study found that the decreased sFNC of FPN-SMN was associated with lower BPF and GMV in RRMS males. Our findings support the hypothesis that male RRMS patients may have a higher degree of static connectivity impairment based on functional network evidence, and these functional reorganizations may be associated with structural damage. Recently, a systematic review found no clear trend towards one FC direction change in MS, which may be associated with the large heterogeneity within and between different study cohorts (e.g., different fMRI indicators, MS phenotypes, disease status, duration, and age) (35). Hence, our results still need to be interpreted cautiously.

### Altered dFNC temporal properties in female patients with RRMS

Three recurring states were identified for dFNC. State 1, a sparse connected state, is characterized by overall lower connectivity within and between networks; it possibly reflects the baseline state of minimal activity between brain neurons at rest. State 2 and State 3 are more tightly connected states within or between the DMN, FPN, SMN, and VIS, which may suggest the active states of cognitive and motor networks aroused by the brain. Results of the dFNC temporal properties showed that female RRMS patients exhibited a higher fraction time and more dwell time with lower transitions in State 1 rather than State 2 usually exhibited among healthy females, suggesting that RRMS could cause the transition from the active state of cognitive networks (State 2) to the baseline state (State 1) with sparse connectivity in female patients. These results are supported by a recent longitudinal study, which found decreased dFNC even in clinically isolated syndromes, and the reduced dynamics were more significant over time. Importantly, the functional connectivity within a tightly connected state at baseline could predict cognitive performance after 5 years in MS (36).

Moreover, further ROC analysis indicted that the fraction time and mean dwell time of State 1 could discriminate female patients with RRMS from healthy females. The sparse connected state (State 1) is the most frequent dFNC state for RRMS patients, which is consistent with the findings of Hidalgo et al., which also observed that the decreased dFNC of State 1 was associated with poor motor and cognitive performance in MS patients (37). Our results indicated that the temporal properties of State 1 may be potential neuroimaging markers in female patients. Interestingly, the lower fraction and dwell time of State 1 related to lower GMV, and the higher numbers of transitions related to gray and white matter atrophy. These findings may suggest that female patients still trend toward dynamic functional compensation in response to structural damage in the early stages of disease, even if such functional compensation is unsuccessful.

### Significant dFNC decreases in female patients with RRMS

Dynamic reorganization was only seen in female RRMS patients: reduced connectivity mainly within the DMN, FPN, and VIS in all states. These findings are consistent with a recent study into dynamic eigenvector centrality, which revealed that cognitively impaired MS patients had decreased dynamics in the DMN, FPN, and VIS compared with healthy controls (38). Our results further indicated that female patients not only show the transition of cognitive-related networks (from State 2 to State 1), but also decreased dFNC within the cognitive networks. As such, we speculate that altered dFNC temporal properties and decreased dFNC within the cognition networks may be maladaptive approaches to maintaining functioning in female patients.

However, the mechanism of how RRMS affects functional networks by sex is still unclear. It may be associated with the complex interactions of sex hormone levels, regulation of the immune system, and certain MS susceptibility genes (39–41). Studies have suggested that sex hormones may have beneficial effects on reducing inflammation and neurodegeneration in MS, which were confirmed in mouse models of MS/demyelination (42, 43). Although a previous study has reported gray matter loss associated with ovarian aging in MS (44), there is still a lack of research on how sex hormones affect functional network connectivity in MS patients. Moreover, recent studies have shown that sex is an important regulator of functional network reorganization in both healthy people and patients with Alzheimer’s disease (45). Exploring sex-related differences in functional connectivity could provide important information to characterize the brain and cognitive changes of RRMS patients.

Our study is not without limitations. It is still preliminary work and may be affected by different disease durations, disabilities, and disease states. Thus, further research should be carried out based on these points to verify our results. Although our results revealed that female RRMS patients showed reduced dFNC on cognition-related networks, the lack of cognitive-related assessments limited our interpretation of cognitive-related networks (e.g., DMN) due to the retrospective design.

In conclusion, our study found that RRMS affected static connectivity in males and dynamic connectivity in females, suggesting that sex-related effects may be important factors for functional damage and reorganization of the CNS in RRMS patients. Exploring these sex-related differences might increase the possibility of sex-specific approaches to treating RRMS in the future.

## Data availability statement

The raw data supporting the conclusions of this article will be made available by the authors, without undue reservation.

## Ethics statement

The studies involving human participants were reviewed and approved by Ethics Committee of The First Affiliated Hospital of Nanchang University. The patients/participants provided their written informed consent to participate in this study. Written informed consent was obtained from the individual(s) for the publication of any potentially identifiable images or data included in this article.

## Author contributions

YW, FZ, YXL, and YD contributed to drafting/revising the manuscript. YLW contributed to data analysis. ZZ, NZ, XH, and CZ performed the statistical analysis. XC, MH, YZ, HL, and GC contributed to MRI data acquisition and analysis. JS and YML contributed to interpretation of the data. All authors approved and take public responsibility for the version to be published.

## Supplementary material

Supplementary Figure 1 Receiver operating characteristic curve of dFNC temporal properties. AUC, areas under the curve.

Supplementary Figure 2 Significant results of voxel-level comparison of RSN maps and sFNC between groups with gray matter volume as a covariate. Spatial maps of significant voxels **(A1)** and sFNC **(A2)** of RSNs in healthy groups, *P*<0.05, FDR corrected. Spatial maps of significant voxels **(B1)** and sFNC **(B2)** of RSNs between RRMS males and healthy males, *P*<0.05, FDR corrected. Significant cluster volume>10. RRMS, relapsing-remitting multiple sclerosis; DMN, default mode network; SMN, sensorimotor network; VIS, visual network; FPN, frontoparietal network; DAN, dorsal attention network; VAN, ventral attention network.

Supplementary Figure 3 Results of clustering analysis and between-groups dynamic temporal properties with gray matter volume as a covariate. **(A)** Three dFNC states for all subjects with the total number of occurrences and percentage of total occurrences. **(B)** The specific connectivity state for each group. The percentage is calculated as the ratio of the number of subjects who entered one state to the total number of subjects in each group. **(C)** Significant differences in the temporal properties in healthy controls and RRMS groups: healthy females showed a higher fraction time (*P*<0.001) and more dwell time in State 2 (*P*<0.001) with lower transitions (*P*<0.001) compared with healthy males, while female RRMS patients exhibited a higher fraction time (*P*<0.001) and more dwell time in State 1 (*P*<0.001) and higher transitions (*P*=0.005) relative to healthy females. Fraction time: the total time percentage of one subject staying in a state. Mean dwell time: the time each subject spent in a specific state. Transition number: the total number of subject transitions from one state to another. *** indicates *P*<0.001, ** means *P*<0.01, * means *P*<0.05. RRMS, relapsing-remitting multiple sclerosis; DMN, default mode network; SMN, sensorimotor network; VIS, visual network; FPN, frontoparietal network; DAN, dorsal attention network; VAN, ventral attention network; BG, basal ganglia network.

Supplementary Figure 4 Significant differences in dFNC between groups with gray matter volume as a covariate. **(A)** Healthy females showed significantly higher dFNC within DMN, PFN, and VIS compared with healthy males (*P*<0.05, FDR corrected). **(B)** RRMS females showed significantly lower dFNC within DMN, PFN, and VIS compared with healthy females (*P*<0.05, FDR corrected). RRMS, relapsing-remitting multiple sclerosis; DMN, default mode network; SMN, sensorimotor network; VIS, visual network; FPN, frontoparietal network; DAN, dorsal attention network; VAN, ventral attention network; BG, basal ganglia network.

Supplementary Figure 5 Results of clustering analysis and between-groups dynamic temporal properties when the window width was 22 TRs, the step was 1 TR, and the number of clusters was four. **(A)** Four dFNC states for all subjects with the total number of occurrences and percentage of total occurrences. **(B)** The specific connectivity state for each group. The percentage is calculated as the ratio of the number of subjects who entered one state to the total number of subjects in each group. **(C)** Significant differences in the temporal properties in healthy controls and RRMS groups: healthy females showed a higher fraction time (*P*<0.001) and more dwell time in State 2 (*P*<0.001) with lower transitions (*P*<0.05) compared with healthy males, while female RRMS patients exhibited a higher fraction time (*P*<0.001) and more dwell time in State 1 (*P*<0.001) and higher transitions (*P*=0.005) relative to healthy females. Fraction time: the total time percentage of one subject staying in a state. Mean dwell time: the time each subject spent in a specific state. Transition number: the total number of subject transitions from one state to another. *** indicates *P*<0.001, ** means *P*<0.01, * indicates *P*<0.05. RRMS, relapsing-remitting multiple sclerosis; DMN, default mode network; SMN, sensorimotor network; VIS, visual network; FPN, frontoparietal network; DAN: dorsal attention network; VAN, ventral attention network; BG, basal ganglia network.

Supplementary Figure 6 Results of clustering analysis and between-groups dynamic temporal properties when the window width was 30 TRs, the step was 1 TR, and the number of clusters was three. **(A)** Three dFNC states for all subjects with the total number of occurrences and percentage of total occurrences. **(B)** The specific connectivity state for each group. The percentage is calculated as the ratio of the number of subjects who entered one state to the total number of subjects in each group. **(C)** Significant differences in the temporal properties in healthy controls and RRMS groups: healthy females showed a higher fraction time (*P*<0.001) and more dwell time in State 2 (*P*<0.001) compared with healthy males, while female RRMS patients exhibited a higher fraction time (*P*<0.001) and more dwell time in State 1 (*P*<0.001) and higher transitions (*P*=0.01) relative to healthy females. Fraction time: the total time percentage of one subject staying in a state. Mean dwell time: the time each subject spent in a specific state. Transition number: the total number of subject transitions from one state to another. *** indicates *P*<0.001, ** means *P*<0.01. RRMS, relapsing-remitting multiple sclerosis; DMN, default mode network; SMN, sensorimotor network; VIS, visual network; FPN, frontoparietal network; DAN: dorsal attention network; VAN, ventral attention network; BG, basal ganglia network.

Supplementary Figure 7 Results of clustering analysis and between-groups dynamic temporal properties when the window width was 30 TRs, the step was 1 TR, and the number of clusters was four. **(A)** Four dFNC states for all subjects with the total number of occurrences and percentage of total occurrences. **(B)** The specific connectivity state for each group. The percentage is calculated as the ratio of the number of subjects who entered one state to the total number of subjects in each group. **(C)** Significant differences in the temporal properties in healthy controls and RRMS groups: healthy females showed a higher fraction time (*P*<0.001) and more dwell time in State 2 (*P*<0.001) compared with healthy males, while female RRMS patients exhibited a higher fraction time (*P*<0.001) and more dwell time in State 1 (*P*<0.001) and higher transitions (*P*=0.002) relative to healthy females. Fraction time: the total time percentage of one subject staying in a state. Mean dwell time: the time each subject spent in a specific state. Transition number: the total number of subject transitions from one state to another. *** indicates *P*<0.001, ** means *P*<0.01, * indicates *P*<0.05. RRMS, relapsing-remitting multiple sclerosis; DMN, default mode network; SMN, sensorimotor network; VIS, visual network; FPN, frontoparietal network; DAN: dorsal attention network; VAN, ventral attention network; BG, basal ganglia network.

Supplementary Figure 8 Significant differences in dFNC between groups when the window width was 22 TRs, the step was 1 TR, and the number of clusters was four. **(A)** Healthy females showed significantly higher dFNC within DMN, PFN, and VIS compared with healthy males (*P*<0.05, FDR corrected). **(B)** RRMS females showed significantly lower dFNC within DMN, PFN, and VIS compared with healthy females (*P*<0.05, FDR corrected). RRMS, relapsing-remitting multiple sclerosis; DMN, default mode network; SMN, sensorimotor network; VIS, visual network; FPN, frontoparietal network; DAN: dorsal attention network; VAN, ventral attention network; BG, basal ganglia network.

Supplementary Figure 9 Significant differences in dFNC between groups when the window width was 30 TRs, the step was 1 TR, and the number of clusters was three. **(A)** Healthy females showed significantly higher dFNC within DMN, PFN, and VIS compared with healthy males (*P*<0.05, FDR corrected). **(B)** RRMS females showed significantly lower dFNC within DMN, PFN, and VIS compared with healthy females (*P*<0.05, FDR corrected). RRMS, relapsing-remitting multiple sclerosis; DMN, default mode network; SMN, sensorimotor network; VIS, visual network; FPN, frontoparietal network; DAN: dorsal attention network; VAN, ventral attention network; BG, basal ganglia network.

Supplementary Figure 10 Significant differences in dFNC between groups when the window width was 30 TRs, the step was 1 TR, and the number of clusters was four. **(A)** Healthy females showed significantly higher dFNC within DMN, PFN, and VIS compared with healthy males (*P*<0.05, FDR corrected). **(B)** RRMS females showed significantly lower dFNC within DMN, PFN, and VIS compared with healthy females (*P*<0.05, FDR corrected). RRMS, relapsing-remitting multiple sclerosis; DMN, default mode network; SMN, sensorimotor network; VIS, visual network; FPN, frontoparietal network; DAN, dorsal attention network; VAN, ventral attention network; BG, basal ganglia network.
