# Supplementary figures and images for: Male and female are not the same: a multicenter study of static and dynamic functional connectivity in relapse-remitting multiple sclerosis in China
Source: Front Immunol. 2023 Oct 10;14:1216310. doi: 10.3389/fimmu.2023.1216310 (PMC10597802; doi:10.3389/fimmu.2023.1216310)

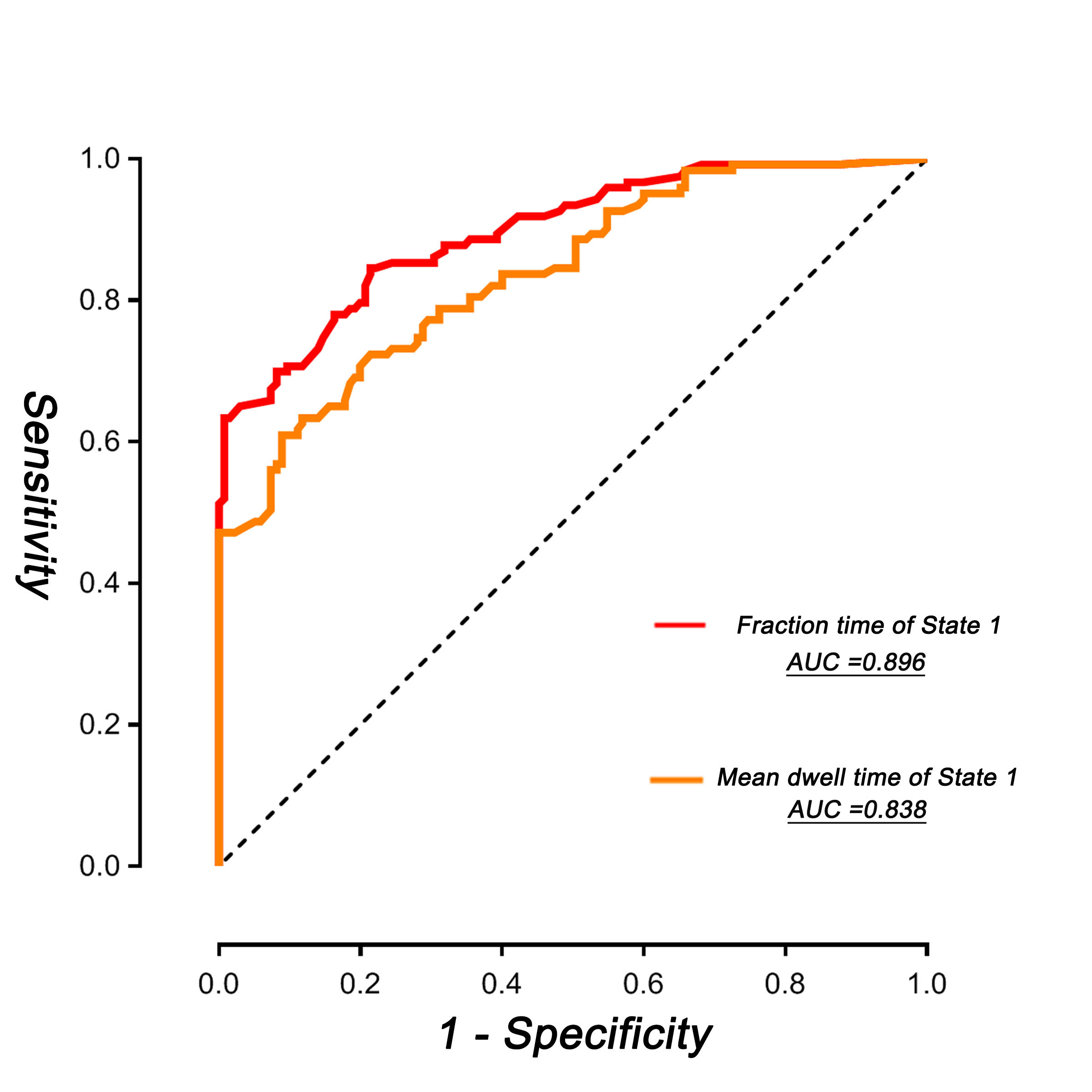

Supplement: Supplementary Figure 1 — Receiver operating characteristic curve of dFNC temporal properties. AUC, areas under the curve. [file Image_1.jpeg]

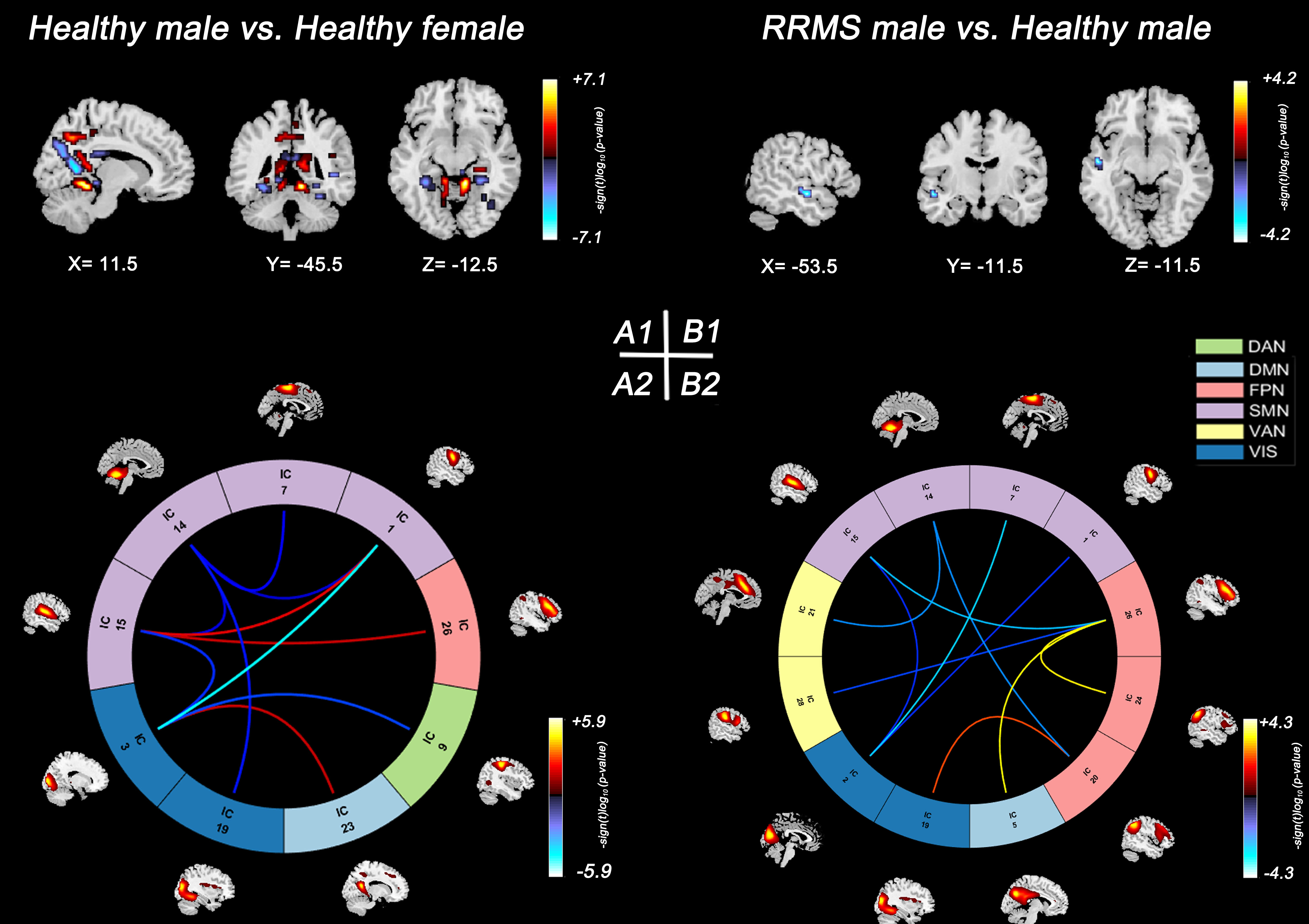

Supplement: Supplementary Figure 2 — Significant results of voxel-level comparison of RSN maps and sFNC between groups with gray matter volume as a covariate. Spatial maps of significant voxels (A1) and sFNC (A2) of RSNs in healthy groups, P<0.05, FDR corrected. Spatial maps of significant voxels (B1) and sFNC (B2) of RSNs between RRMS males and healthy males, P<0.05, FDR corrected. Significant cluster volume>10. RRMS, relapsing-remitting multiple sclerosis; DMN, default mode network; SMN, sensorimotor network; VIS, visual network; FPN, frontoparietal network; DAN, dorsal attention network; VAN, ventral attention network. [file Image_2.jpeg]

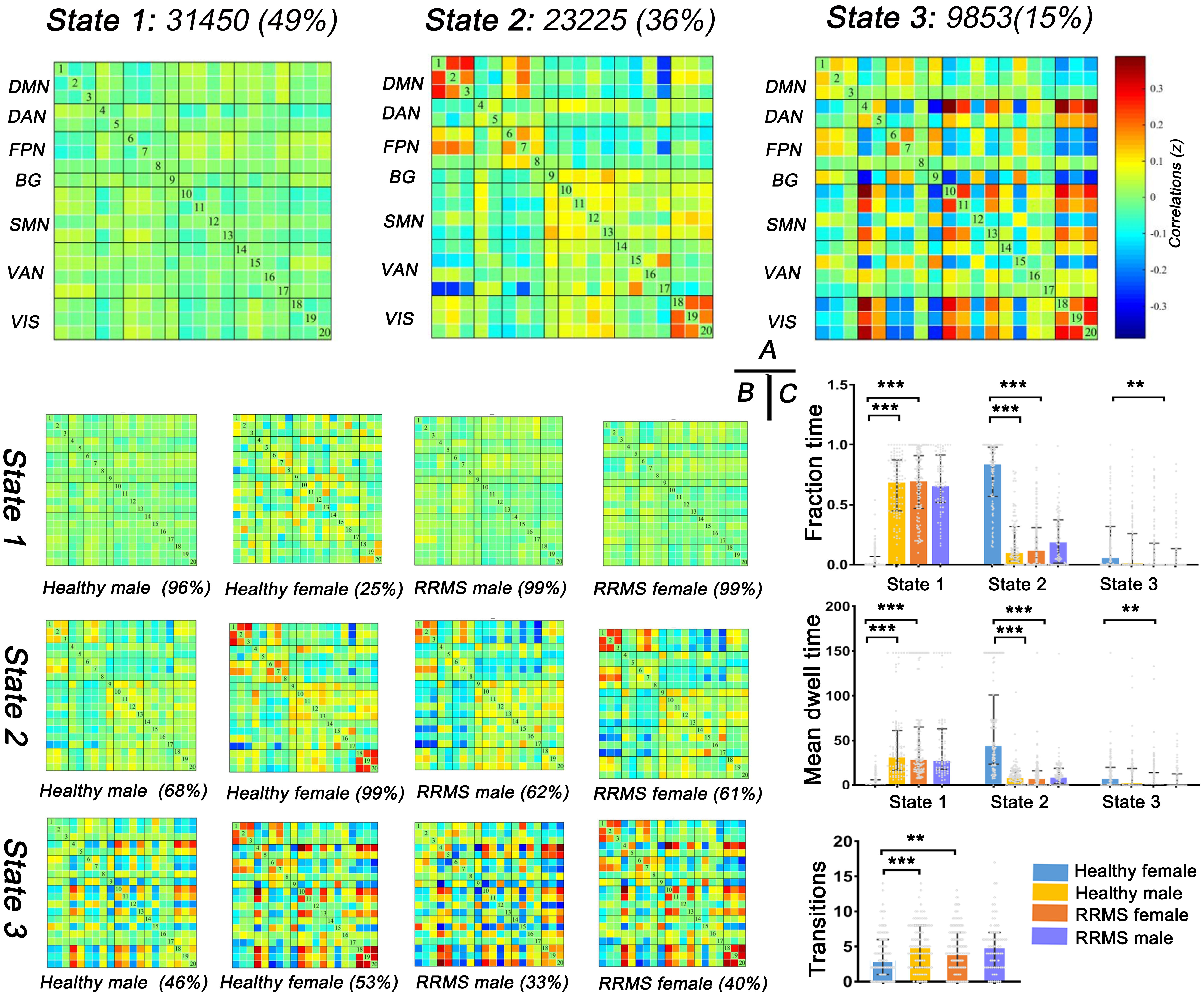

Supplement: Supplementary Figure 3 — Results of clustering analysis and between-groups dynamic temporal properties with gray matter volume as a covariate. (A) Three dFNC states for all subjects with the total number of occurrences and percentage of total occurrences. (B) The specific connectivity state for each group. The percentage is calculated as the ratio of the number of subjects who entered one state to the total number of subjects in each group. (C) Significant differences in the temporal properties in healthy controls and RRMS groups: healthy females showed a higher fraction time (P<0.001) and more dwell time in State 2 (P<0.001) with lower transitions (P<0.001) compared with healthy males, while female RRMS patients exhibited a higher fraction time (P<0.001) and more dwell time in State 1 (P<0.001) and higher transitions (P=0.005) relative to healthy females. Fraction time: the total time percentage of one subject staying in a state. Mean dwell time: the time each subject spent in a specific state. Transition number: the total number of subject transitions from one state to another. *** indicates P<0.001, ** means P<0.01, * means P<0.05. RRMS, relapsing-remitting multiple sclerosis; DMN, default mode network; SMN, sensorimotor network; VIS, visual network; FPN, frontoparietal network; DAN, dorsal attention network; VAN, ventral attention network; BG, basal ganglia network. [file Image_3.jpeg]

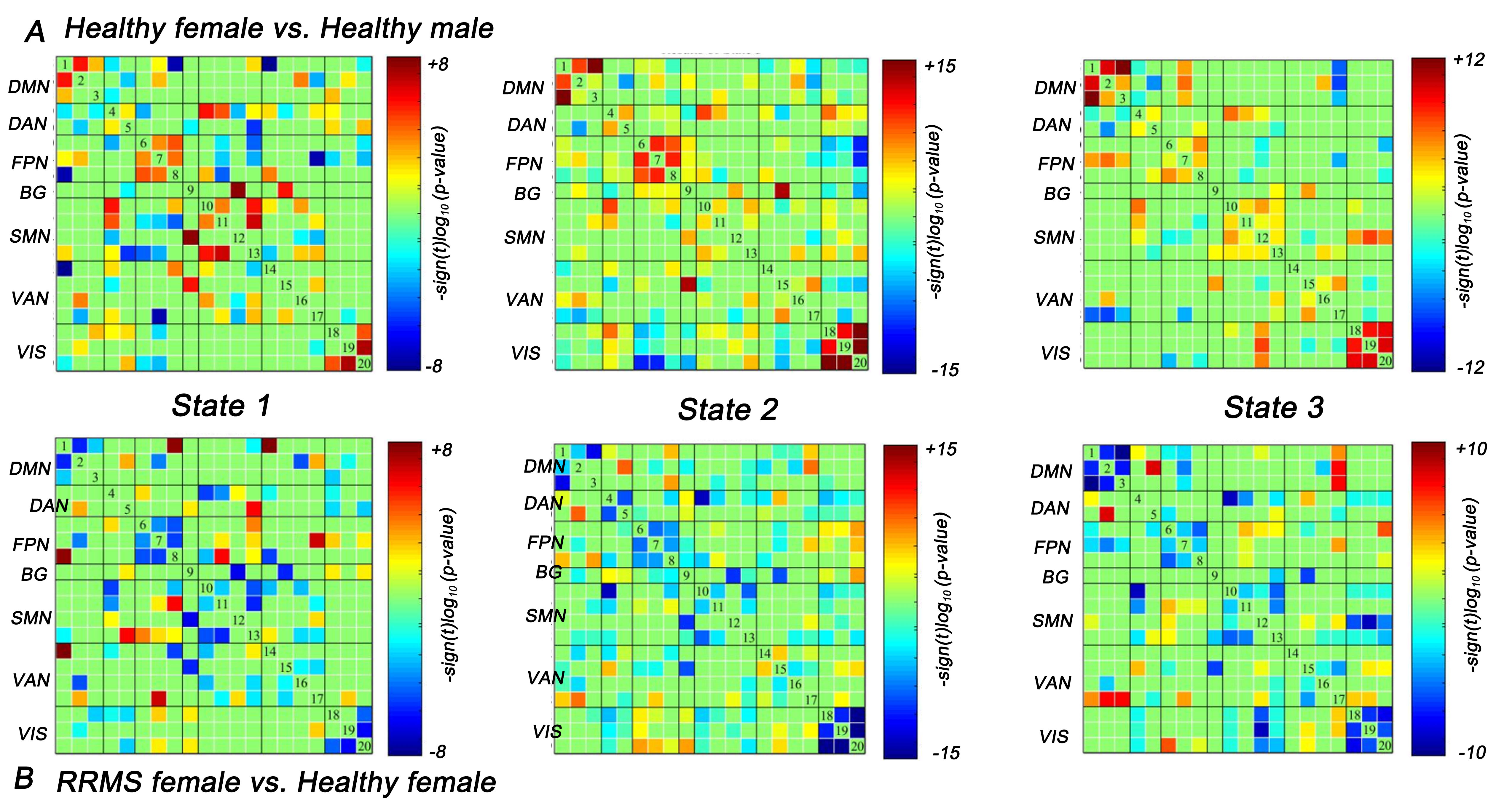

Supplement: Supplementary Figure 4 — Significant differences in dFNC between groups with gray matter volume as a covariate. (A) Healthy females showed significantly higher dFNC within DMN, PFN, and VIS compared with healthy males (P<0.05, FDR corrected). (B) RRMS females showed significantly lower dFNC within DMN, PFN, and VIS compared with healthy females (P<0.05, FDR corrected). RRMS, relapsing-remitting multiple sclerosis; DMN, default mode network; SMN, sensorimotor network; VIS, visual network; FPN, frontoparietal network; DAN, dorsal attention network; VAN, ventral attention network; BG, basal ganglia network. [file Image_4.jpeg]

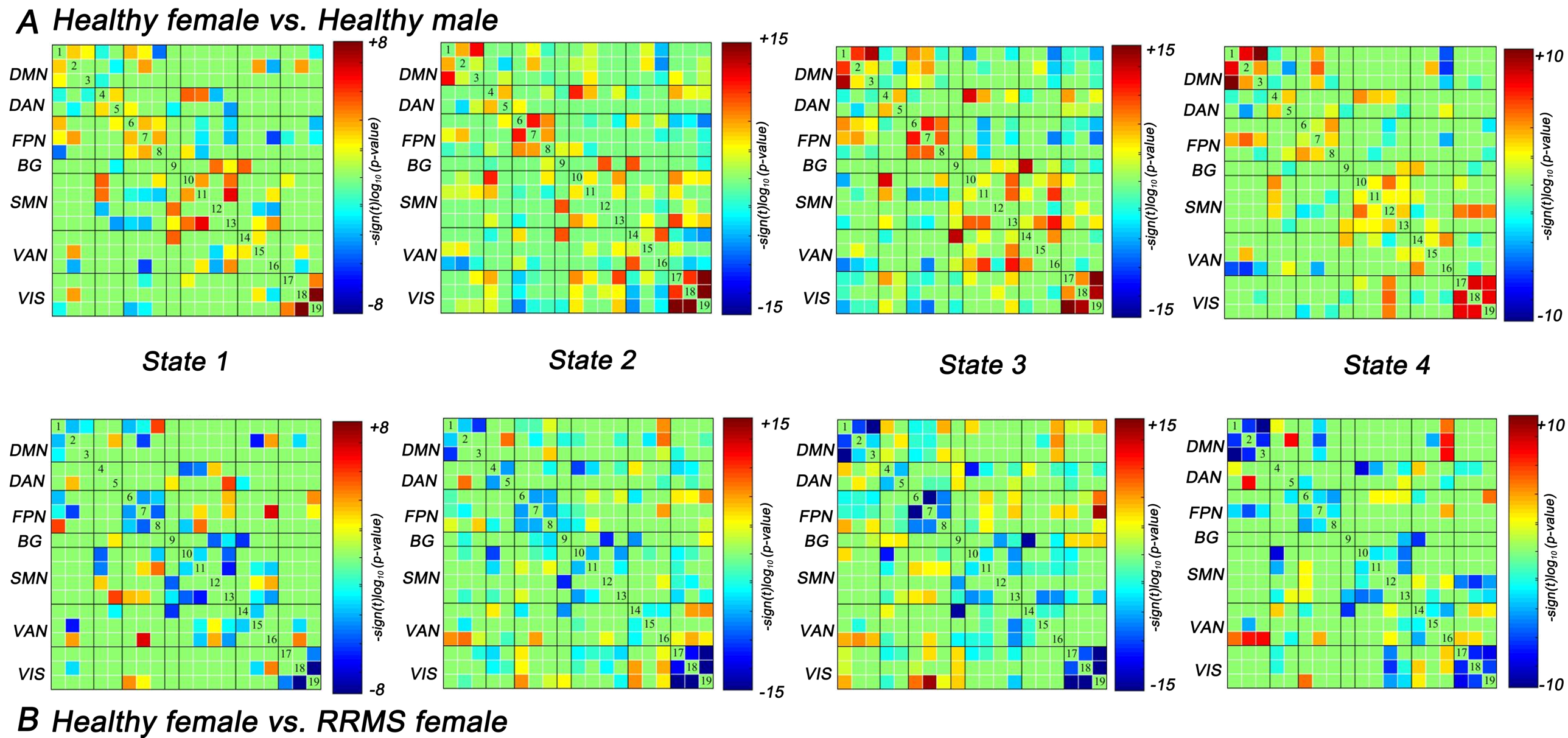

Supplement: Supplementary Figure 8 — Significant differences in dFNC between groups when the window width was 22 TRs, the step was 1 TR, and the number of clusters was four. (A) Healthy females showed significantly higher dFNC within DMN, PFN, and VIS compared with healthy males (P<0.05, FDR corrected). (B) RRMS females showed significantly lower dFNC within DMN, PFN, and VIS compared with healthy females (P<0.05, FDR corrected). RRMS, relapsing-remitting multiple sclerosis; DMN, default mode network; SMN, sensorimotor network; VIS, visual network; FPN, frontoparietal network; DAN: dorsal attention network; VAN, ventral attention network; BG, basal ganglia network. [file Image_8.jpeg]

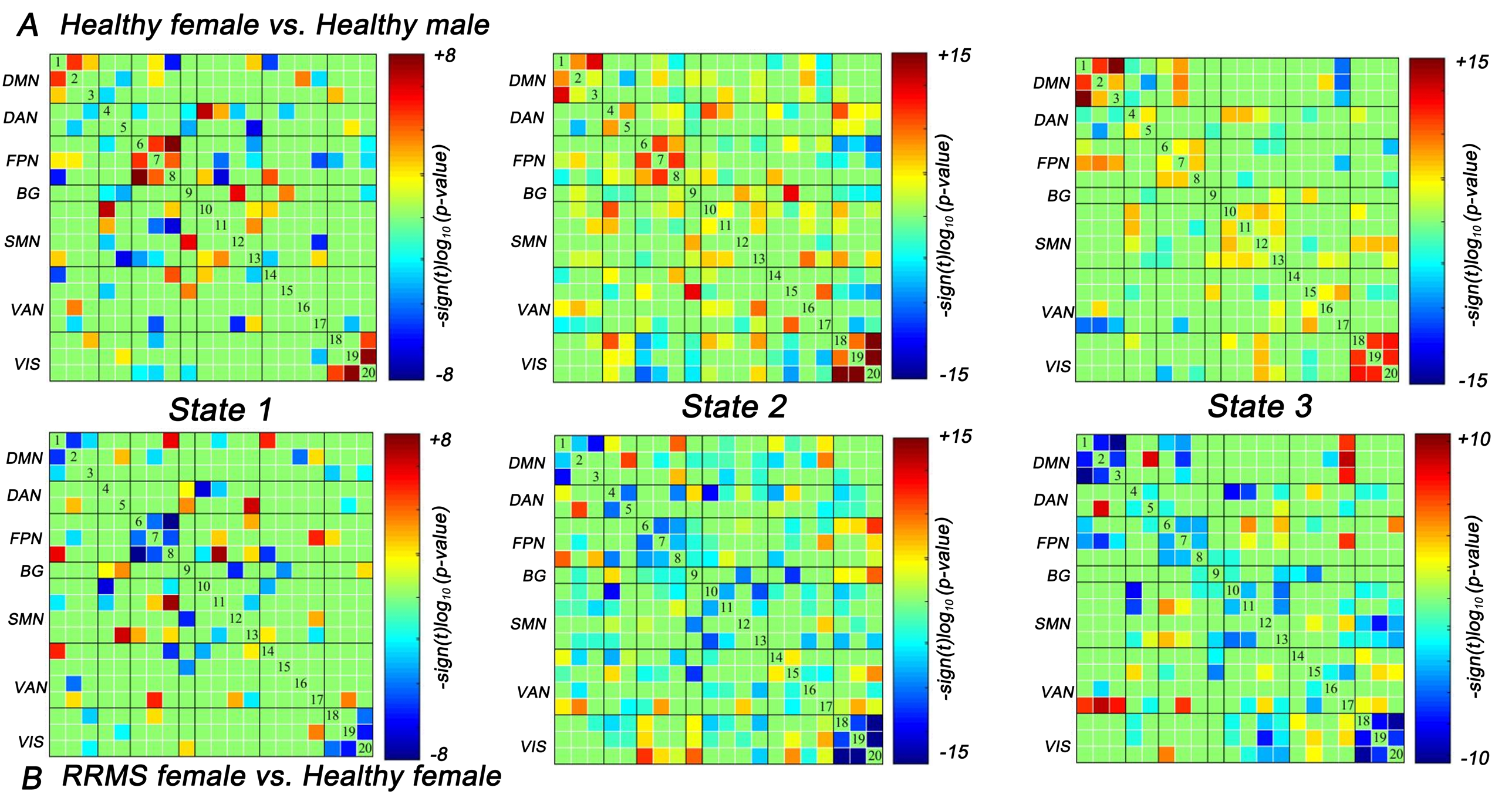

Supplement: Supplementary Figure 9 — Significant differences in dFNC between groups when the window width was 30 TRs, the step was 1 TR, and the number of clusters was three. (A) Healthy females showed significantly higher dFNC within DMN, PFN, and VIS compared with healthy males (P<0.05, FDR corrected). (B) RRMS females showed significantly lower dFNC within DMN, PFN, and VIS compared with healthy females (P<0.05, FDR corrected). RRMS, relapsing-remitting multiple sclerosis; DMN, default mode network; SMN, sensorimotor network; VIS, visual network; FPN, frontoparietal network; DAN: dorsal attention network; VAN, ventral attention network; BG, basal ganglia network. [file Image_9.jpeg]

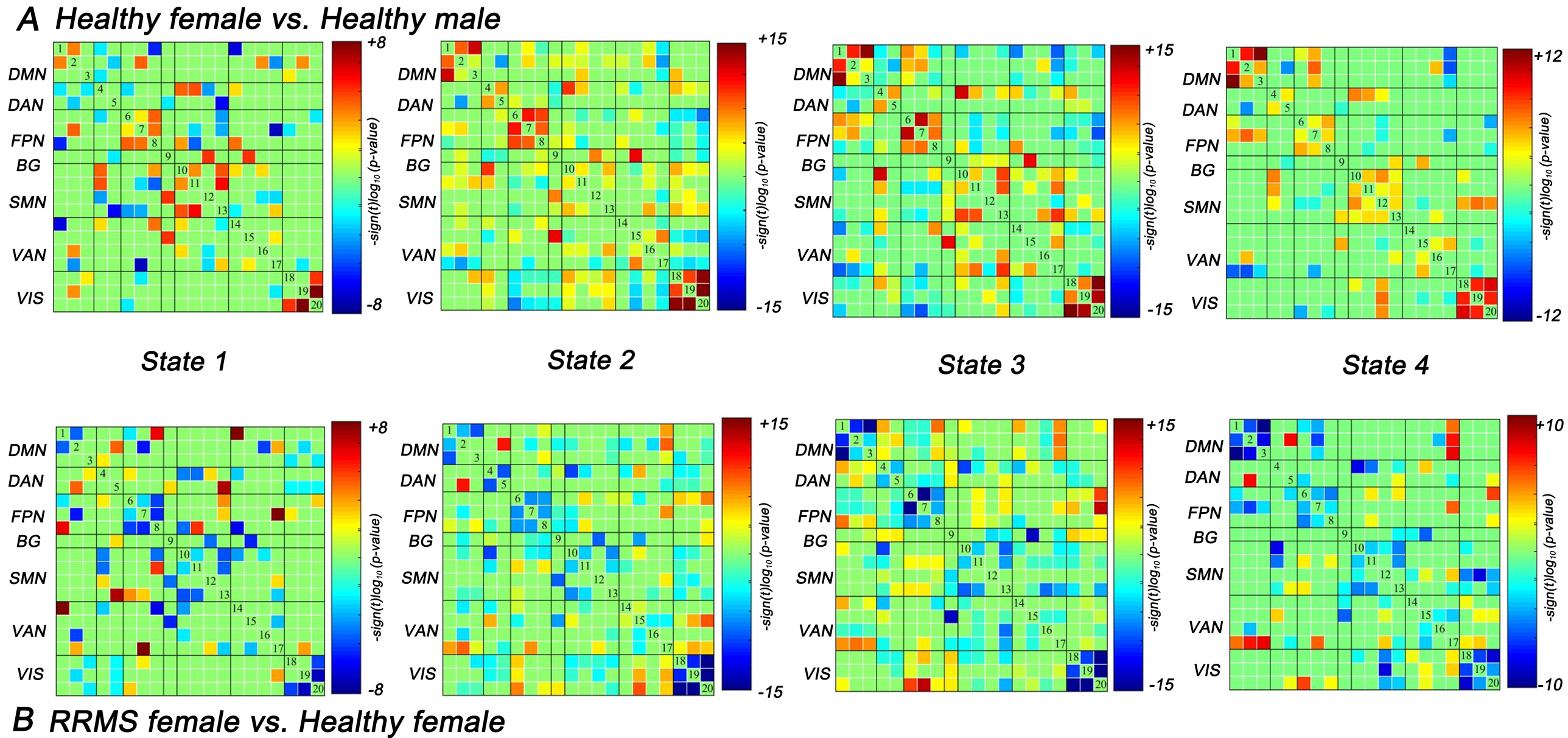

Supplement: Supplementary Figure 10 — Significant differences in dFNC between groups when the window width was 30 TRs, the step was 1 TR, and the number of clusters was four. (A) Healthy females showed significantly higher dFNC within DMN, PFN, and VIS compared with healthy males (P<0.05, FDR corrected). (B) RRMS females showed significantly lower dFNC within DMN, PFN, and VIS compared with healthy females (P<0.05, FDR corrected). RRMS, relapsing-remitting multiple sclerosis; DMN, default mode network; SMN, sensorimotor network; VIS, visual network; FPN, frontoparietal network; DAN, dorsal attention network; VAN, ventral attention network; BG, basal ganglia network. [file Image_10.jpeg]
