# Supplementary material for: Male and female are not the same: a multicenter study of static and dynamic functional connectivity in relapse-remitting multiple sclerosis in China
Source: Front Immunol. 2023 Oct 10;14:1216310. doi: 10.3389/fimmu.2023.1216310 (PMC10597802; doi:10.3389/fimmu.2023.1216310)
Supplement: Supplementary file 11 [file Table_1.docx]

**Table S1**. **Peak Coordinates of ICs**

| RSNs | ICs | Brain Regions | Peak Coordinates (x, y, z) |
| --- | --- | --- | --- |
| DMN | 6 | Bilateral Anterior Cingulate Cortex | -0.5, 53.5, 21.5 |
|  | 23 | Bilateral Posterior Cingulate/Bilateral Precuneus | 15.5, -54.5, 17.5 |
|  | 5 | Bilateral Precuneus | -6.5, -68.5, 32.5 |
| DAN | 10 | Bilateral Superior Occipital Gyrus | 23.5, -69.5, 50.5 |
|  | 9 | Bilateral Postcentral gyrus | 42.5, -27.5, 48.5 |
| FPN | 20 | Right Inferior Frontal Gyrus/Right Inferior Parietal Lobule | 48.5, -53.5, 44.5 |
|  | 26 | Bilateral Middle Frontal Gyrus | -45.5, 21.5, 24.5 |
|  | 24 | Left inferior frontal gyrus/Left inferior parietal lobule | -50.5, -56.5, 41.5 |
| BG | 4 | Bilateral Putamen | -24.5, 5.5, 2.5 |
| SMN | 15 | Bilateral Superior Temporal Gyrus | 53.5, -21.5, 9.5 |
|  | 7 | Bilateral Paracentral Lobule | -0.5, -23.5, 60.5 |
|  | 1 | Bilateral Postcentral gyrus | -51.5, -9.5, 30.5 |
|  | 14 | Bilateral Cerebellum (VI) | -2.5, -53.5, -20.5 |
| VAN | 28 | Bilateral supramarginal gyrus | 59.5, -24.5, 27.5 |
|  | 21 | Bilateral Middle Cingulate Cortex | -2.5, 26.5, 32.5 |
|  | 25 | Bilateral Angular Gyrus | 59.5, -47.5, 23.5 |
|  | 12 | Bilateral Supplementary Motor Area | -5.5, 6.5, 54.5 |
| VIS | 2 | Bilateral Calcarine Gyrus | 2.5, -77.5, 14.5 |
|  | 3 | Bilateral Middle Occipital Gyrus | 18.5, -93.5, 2.5 |
|  | 19 | Bilateral Middle Occipital Gyrus | 35.5, -81.5, 12.5 |

ICs, independent components; RSNs, resting-state networks; DMN, default mode network; DAN: dorsal attention network; FPN, frontoparietal network; BG, basal ganglia network; SMN, sensorimotor network; VAN, ventral attention network; VIS, visual network.
