# Supplementary material for: Male and female are not the same: a multicenter study of static and dynamic functional connectivity in relapse-remitting multiple sclerosis in China
Source: Front Immunol. 2023 Oct 10;14:1216310. doi: 10.3389/fimmu.2023.1216310 (PMC10597802; doi:10.3389/fimmu.2023.1216310)
Supplement: Supplementary file 12 [file Table_2.docx]

**Table S2** Results of correlation analysis in female patients with RRMS

| **GMV (ml)^a^** | | ***r*** | ***P*** |
| --- | --- | --- | --- |
|  | EDSS | -0.099 | 0.270 |
|  | Fraction time of State 1 | **0.224** | **0.011** |
|  | Mean dwell time of state 1 | **0.305** | **<0.001** |
|  | Fraction time of State 2 | **-0.23** | **0.009** |
|  | Mean dwell time of state 2 | **-0.209** | **0.018** |
|  | Fraction time of State 3 | -0.129 | 0.149 |
|  | Mean dwell time of state 3 | -0.101 | 0.256 |
|  | Transitions | **-0.314** | **<0.001** |
|  | DD | **-0.224** | **0.011** |
| **WMV (ml)^a^** | | | |
|  | EDSS | -0.012 | 0.089 |
|  | Fraction time of State 1 | 0.134 | 0.132 |
|  | Mean dwell time of state 1 | **0.206** | **0.020** |
|  | Fraction time of State 2 | -0.15 | 0.093 |
|  | Mean dwell time of state 2 | -0.137 | 0.125 |
|  | Fraction time of State 3 | -0.066 | 0.458 |
|  | Mean dwell time of state 3 | -0.057 | 0.522 |
|  | Transitions | **-0.225** | **0.011** |
|  | DD | -0.17 | 0.056 |
| **BPF^a^** | | | |
|  | EDSS | -0.151 | 0.089 |
|  | Fraction time of State 1 | 0.024 | 0.792 |
|  | Mean dwell time of state 1 | 0.079 | 0.376 |
|  | Fraction time of State 2 | -0.087 | 0.329 |
|  | Mean dwell time of state 2 | -0.11 | 0.217 |
|  | Fraction time of State 3 | 0.031 | 0.732 |
|  | Mean dwell time of state 3 | 0.038 | 0.670 |
|  | Transitions | -0.112 | 0.208 |
|  | DD | **-0.313** | **<0.001** |
| **LV (ml)^a^** | | | |
|  | EDSS | **0.194** | **0.029** |
|  | BPF | **-0.503** | **<0.001** |
|  | GMV | **-0.27** | **0.002** |
|  | WMV | **-0.385** | **<0.001** |
|  | Fraction time of State 1 | 0.082 | 0.359 |
|  | Mean dwell time of state 1 | 0.066 | 0.463 |
|  | Fraction time of State 2 | -0.067 | 0.453 |
|  | Mean dwell time of state 2 | -0.056 | 0.535 |
|  | Fraction time of State 3 | -0.086 | 0.336 |
|  | Mean dwell time of state 3 | -0.066 | 0.464 |
|  | Transitions | -0.099 | 0.268 |
|  | DD | **0.291** | **<0.001** |
| **DD (months)^a^** | | | |
|  | Fraction time of State 1 | -0.119 | 0.184 |
|  | Mean dwell time of state 1 | **-0.181** | **0.041** |
|  | Fraction time of State 2 | 0.072 | 0.423 |
|  | Mean dwell time of state 2 | 0.041 | 0.646 |
|  | Fraction time of State 3 | 0.103 | 0.249 |
|  | Mean dwell time of state 3 | 0.105 | 0.238 |
|  | Transitions | **0.197** | **0.026** |

Note: ^a^ indicates Spearman correlation.

Abbreviations: RRMS, relapsing-remitting multiple sclerosis; DD, disease duration; EDSS, Extended Disability Status Scale; LV, Lesion volume; GMV, gray matter volume; WMV, white matter volume; BPF, brain parenchyma fraction.
