# Supplementary material for: Male and female are not the same: a multicenter study of static and dynamic functional connectivity in relapse-remitting multiple sclerosis in China
Source: Front Immunol. 2023 Oct 10;14:1216310. doi: 10.3389/fimmu.2023.1216310 (PMC10597802; doi:10.3389/fimmu.2023.1216310)
Supplement: Supplementary file 13 [file Table_3.docx]

**Table S3** Results of correlation analysis in male patients with RRMS

|  | | *r* | *P* |
| --- | --- | --- | --- |
| **GMV (ml)^b^** | | | |
|  | EDSS | **-0.308** | **0.013** |
|  | within FPN (IC24 - IC26) | **-0.251** | **0.044** |
|  | SMN-FPN (IC14 - IC20) | **-0.311** | **0.012** |
|  | FPN-VIS (26-19) | -0.085 | 0.502 |
|  | FPN-VIS (IC20 -IC19) | 0.242 | 0.053 |
|  | SMN-FPN (IC15 - IC26) | -0.023 | 0.853 |
|  | FPN-VAN (IC26 - IC28) | 0.043 | 0.736 |
|  | SMN-VIS (IC2 - IC1) | 0.027 | 0.829 |
|  | SMN-VIS (IC2 - IC7) | 0.229 | 0.067 |
|  | SMN-VAN (IC14 - IC21) | 0.042 | 0.737 |
|  | SMN-VIS (IC15 - IC2) | -0.087 | 0.492 |
|  | DD | **-0.439** | **<0.001** |
| **BPF^b^** | | | |
|  | EDSS | **-0.317** | **0.01** |
|  | within FPN (IC24 - IC26) | -0.15 | 0.232 |
|  | SMN-FPN (IC14 - IC20) | **-0.284** | **0.022** |
|  | FPN-VIS (26-19) | -0.114 | 0.365 |
|  | FPN-VIS (IC20 -IC19) | -0.029 | 0.817 |
|  | SMN-FPN (IC15 - IC26) | -0.002 | 0.995 |
|  | FPN-VAN (IC26 - IC28) | 0.074 | 0.555 |
|  | SMN-VIS (IC2 - IC1) | 0.070 | 0.577 |
|  | SMN-VIS (IC2 - IC7) | 0.085 | 0.502 |
|  | SMN-VAN (IC14 - IC21) | -0.054 | 0.667 |
|  | SMN-VIS (IC15 - IC2) | -0.090 | 0.477 |
|  | DD | **-0.25** | **0.045** |
| **LV (ml)^a^** | | | |
|  | EDSS | **0.352** | **0.004** |
|  | BPF | **-0.314** | **0.011** |
|  | within FPN (IC24 - IC26) | -0.154 | 0.222 |
|  | SMN-FPN (IC14 - IC20) | 0.072 | 0.568 |
|  | FPN-VIS (26-19) | -0.097 | 0.444 |
|  | FPN-VIS (IC20 -IC19) | -0.071 | 0.576 |
|  | SMN-FPN (IC15 - IC26) | -0.164 | 0.191 |
|  | FPN-VAN (IC26 - IC28) | 0.059 | 0.640 |
|  | SMN-VIS (IC2 - IC1) | -0.098 | 0.439 |
|  | SMN-VIS (IC2 - IC7) | 0.059 | 0.641 |
|  | SMN-VAN (IC14 - IC21) | -0.109 | 0.386 |
|  | SMN-VIS (IC15 - IC2) | 0.102 | 0.419 |
|  | DD | **0.432** | **<0.001** |
| **WMV (ml)^a^** | | | |
|  | EDSS | **-0.338** | **0.006** |
|  | within FPN (IC24 - IC26) | -0.087 | 0.491 |
|  | SMN-FPN (IC14 - IC20) | -0.22 | 0.079 |
|  | FPN-VIS (IC26 - IC19) | -0.034 | 0.788 |
|  | FPN-VIS (IC20 -IC19) | 0.199 | 0.111 |
|  | SMN-FPN (IC15 - IC26) | -0.002 | 0.990 |
|  | FPN-VAN (IC26 - IC28) | -0.051 | 0.689 |
|  | SMN-VIS (IC2 - IC1) | -0.106 | 0.401 |
|  | SMN-VIS (IC2 - IC7) | 0.144 | 0.252 |
|  | SMN-VAN (IC14 - IC21) | 0.143 | 0.257 |
|  | SMN-VIS (IC15 - IC2) | -0.126 | 0.318 |
|  | DD | **-0.328** | **0.008** |
| **DD (months)a** | | | |
|  | EDSS | **0.368** | **0.001** |
|  | within FPN (IC24 - IC26) | -0.12 | 0.343 |
|  | SMN-FPN (IC14 - IC20) | **0.296** | **0.017** |
|  | FPN-VIS (IC26 - IC19) | -0.074 | 0.558 |
|  | FPN-VIS (IC20 -IC19) | 0.084 | 0.506 |
|  | SMN-FPN (IC15 - IC26) | 0.075 | 0.552 |
|  | FPN-VAN (IC26 - IC28) | 0.202 | 0.107 |
|  | SMN-VIS (IC2 - IC1) | -0.169 | 0.107 |
|  | SMN-VIS (IC2 - IC7) | -0.035 | 0.781 |
|  | SMN-VAN (IC14 - IC21) | 0.018 | 0.888 |
|  | SMN-VIS (IC15 - IC2) | -0.057 | 0.650 |

Note: ^a^ indicates spearman correlation, ^b^ indicates partial correlation.

Abbreviations: RRMS, relapsing-remitting multiple sclerosis; DD, disease duration; EDSS, Extended Disability Status Scale; LV, Lesion volume; GMV, gray matter volume; WMV, white matter volume; BPF, brain parenchyma fraction; IC, independent component.

IC1, bilateral postcentral gyrus; IC2, bilateral calcarine gyrus; IC7, bilateral paracentral lobule; IC14, bilateral cerebellum (VI); IC15, bilateral superior temporal gyrus; IC19, bilateral middle occipital gyrus; IC20, right inferior frontal gyrus/right inferior parietal lobule; IC21, bilateral middle cingulate cortex; IC24, left inferior frontal gyrus/left inferior parietal lobule; C26, bilateral middle frontal gyrus; IC28, bilateral postcentral gyrus.
