# Supplementary material for: Male and female are not the same: a multicenter study of static and dynamic functional connectivity in relapse-remitting multiple sclerosis in China
Source: Front Immunol. 2023 Oct 10;14:1216310. doi: 10.3389/fimmu.2023.1216310 (PMC10597802; doi:10.3389/fimmu.2023.1216310)
Supplement: Supplementary file 14 [file DataSheet_1.docx]

ROC curve analysis found that fraction time and mean dwell time of State 1 could significantly distinguish female patients from controls. When the fractional time in State 1 was >0.445, the sensitivity is 78.6%, specificity is 84.6%, and the AUC is 0.896 (P <0.0001). When the mean dwell time was >13.17 s, the sensitivity is 91.1%, specificity is 61.0%, and the AUC is 0.838 (P <0.0001).(**Figure S1**). The remaining indicators are not shown due to the AUC<0.7.
