# Supplementary material for: Male and female are not the same: a multicenter study of static and dynamic functional connectivity in relapse-remitting multiple sclerosis in China
Source: Front Immunol. 2023 Oct 10;14:1216310. doi: 10.3389/fimmu.2023.1216310 (PMC10597802; doi:10.3389/fimmu.2023.1216310)
Supplement: Supplementary file 15 [file DataSheet_2.docx]

**Results**

***Static functional network analysis***

***Intra-network SMs and inter-network sFNC***

In healthy controls, healthy male showed lower SMs within the DMN (bilateral precuneus) and lower sFNC within the SMN and SMN-VIS compared to healthy females (*P*<0.05, FDR corrected) (**Figures S2A1, A2**). However, this sex-difference disappeared between female and male patients with RRMS. Compared to healthy controls, only RRMS males exhibited decreased SMs within DAN, increased sFNC within PFN and reduced sFNC of SMN-PFN, SMN-VIS and SMN-VAN (*P*<0.05, FDR corrected) (**Figures S2B1, B2)**. No significance in RRMS females *vs.* healthy females and RRMS females *vs.* RRMS males.

***Dynamic functional network analysis***

***dFNC clustering states***

First, three recurrent dFNC states were identified after cluster analysis: State 1 (sparse connected state; 49%), State 2 (middle connected state; 36%) and State 3 (high connected state; 15%) (**Figure S3A** ). State 1 was characterized by sparse connectivity both within and between networks, whereas State 3 showed a tightly connected matrix. State 2 was a transitional state between State 1 and State 3, which featured decreased dFNC between the SMN and DMN or FPN, and increased dFNC within the DMN, FPN, SMN and VIS. **Figure S3B** shows the percentage of specific state for each group: healthy females preferred State 2 (99%), whereas healthy males and RRMS patients preferred State 1 (96%, 99%, 99%, respectively).

***dFNC temporal properties***

Next, the temporal properties of dFNC revealed significant differences in fraction time, mean dwell time and transitions among the three states among the groups (**Figure S3C**). Compared to healthy males, healthy females showed higher fraction time (healthy female *vs.* healthy male: 85% *vs.* 11%; *P*<0.001) and more dwell time in State 2 (healthy female *vs.* healthy male: 45.33s *vs.* 4.42s; *P*<0.001) with lower transitions (healthy female *vs.* healthy male: 3 times *vs.* 5 times; *P*<0.001). However, the tendency changed once RRMS was established, female RRMS patients exhibited higher fraction time (RRMS female *vs.* healthy female: 71% *vs.* 0%; *P*<0.001) and more dwell time in State 1 (RRMS female *vs.* healthy female: 30.25s *vs.* 0.00s; *P*<0.001) and higher transitions (RRMS female *vs.* healthy female: 4 times *vs.* 3 times; *P*=0.005), relative to healthy females. No statistical difference was seen in RRMS males *vs.* healthy males and RRMS females *vs.* RRMS males.

***Between-group dFNC differences***

Last, we also evaluated the dFNC differences between the healthy controls and RRMS groups. Similar to the results of the dFNC temporal properties, the dFNC pattern alterations were seen only in female groups: compared to healthy males, healthy females exhibited higher dFNC within DMN, FPN, and VIS in all states (*P*<0.05, FDR corrected) (**Figure S4A**). On the contrary, this trend disappeared among RRMS patients. Moreover, compared to healthy females, female patients mainly showed lower dFNC in all states, particularly within the DMN, FPN, and VIS (*P*<0.05, FDR corrected) (**Figure S4B**). There was no dynamic significance in RRMS males *vs.* healthy males and RRMS females *vs.* RRMS males.
